# Effect of Flow Aids on Mucoadhesive Properties of Polymeric Discs of Polyoxyethylene and Carbopol 971P

**DOI:** 10.4103/0250-474X.62249

**Published:** 2010

**Authors:** M. H. Bele, V. V. Gholap, O. U. Joshi

**Affiliations:** NDMVPS's College of Pharmacy, Department of Pharmaceutics, Shivajinagar, Gangapur Road, Nashik-422 002, India

**Keywords:** Carbopol^®^ 971P, flow aids, mucoadhesion, polyoxyethylene

## Abstract

The aim of present study is to investigate the effect of flow aids on the observed *in vitro* mucoadhesion of two representative polymers; polyoxyethylene and Carbopol^®^ 971P. More recently it has been shown that the addition of small amounts of certain excipients to a mucoadhesive formulation can lead to a substantial decrease in observed mucoadhesion in an *in vitro* test system, which suggests that formulation of these systems could be crucial in developing successful dosage forms. A series of experiments has been carried out which indicates that the presence of flow aids at high concentrations present in tablets can affect the observed *ex-vivo* mucoadhesive bond. Magnesium stearate (5%) exerts its negative effect on the mucoadhesion of Carbopol^®^ 971P and polyoxyethylene combination by hindering the hydration of the polymer. Adhesion time of formulation containing 5% magnesium stearate was found 4.7±0.34 h and percent hydration of same formulation was 70.12%.Talc and colloidal silicon dioxide (Aerosil), which do not possess the same hydrophobic properties or have available divalent cations were found to be viable alternatives to magnesium stearate.

Method dependent parameters such as pH, gastric mucosa, temperature, shape of tablet and dissolution medium influence the mucoadhesion in a particular test system[1]. In addition the physical properties of the polymers used in preparing the mucoadhesive systems have been found to have a significant influence on the observed mucoadhesion[2]. To date, only some limited studies have been carried out on the optimization of mucoadhesive formulations. However, no systematic studies in this area have been published[3–5]. More recently, it has been shown that the addition of small amounts of excipients in tablet formulation can affect observed mucoadhesion in an *in vitro* test system, which suggests that formulation of these systems could be crucial in developing successful dosage forms[6,7].

Polyoxyethylene and Carbopol^®^ 971P were obtained as a gift sample from Colorcon, India Ltd, Goa. Flow aids like magnesium stearate, talc, aerosil (colloidal silicon dioxide) were obtained as a gift sample from Glenmark Pharmaceuticals Ltd., Sinnar, India. All other reagents used were of analytical grade.

Disks (320 mg) of polyoxyethylene, Carbopol^®^ 971P and flow aids were compressed using 16 station rotary tablet compression machine (Rimek) using punch 15×6.5 mm. Hardness of all the disks was maintained in between 65-70 N (Table 1).

**TABLE 1:** FORMULATION OF POLYMERIC DISCS

Formulation	Polyoxyethylene (mg)	Carbopol 971p (mg)	Magnesium Stearate (mg)	Talc (mg)	Aerosil (mg)	Total (mg)
A (2%)	156.8	156.8	6.4	-	-	320
B (5%)	152	152	16	-	-	320
C (2%)	156.8	156.8	-	6.4	-	320
D (5%)	152	152	-	16	-	320
E (2%)	156.8	156.8	-	-	6.4	320
F (5%)	152	152	-	-	16	320
G (Plain)	160	160	-	-	-	320

Adhesion time of polymeric discs was determined by using rotating cylinder method USP type VI apparatus (Disso Lab, India) at 37±0.5° at 100 rpm using 0.1N HCl as a medium[1]. The goat gastric mucosa was adhered to the cylinder by using cynoacrylate glue. The disk was pressed on the mucosa gently with the finger for 1 min. Time upto which disk remains adhered to mucosa was measured as shown in Table 2.

**TABLE 2:** EFFECT OF FLOW AIDS ON ADHESION TIME AND PERCENT HYDRATION

Flow aids	Adhesion Time (h±SD)		Percent hydration	
		
	2%	5%	2%	5%
Magnesium stearate	8.0±1.2	4.7±0.34	75.35	70.12
Talc	8.5±1.7	8.4±0.45	76.56	75.86
Aerosil	8.4±0.6	8.3±1.43	77.45	76.12
Plain tablet	8.6±0.34		78.34	

Swelling index or % of hydration= ((W2−W1)/W2)×100, where, W1 is the initial weight of tablet, W2 is the weight of disks at 12 h. Swelling study of individual polymers and combinations was carried out using rotating paddle at 100 rpm (USP type II dissolution apparatus, Disso 2000 Labindia) and 0.1 N HCl as medium, temperature was maintained at 37±0.5°. The disk was adhered to goat gastric mucosa which was attached to the paddle using cynoacrylate glue and the weights of disks were noted at 1 h intervals (W2).

From the One way ANOVA test it is observed that magnesium stearate, when used in 5% concentration, exhibits strong hydrophobic character and exerts its negative effect on the mucoadhesion of Carbopol^®^ 971P and polyoxyethylene combination by hindering the hydration of the polymer, consequently causing a decrease in the number of carboxylic acid moieties available for bonding to the mucus. The material may also block the passage of water from mucus to the mucoadhesive, which normally occurs by the mechanism of mucus dehydration. In addition it is known that divalent cations can crosslink polyacrylic acids and this may contribute to the deleterious effects that the high concentration of magnesium stearate has on the observed mucoadhesion. It is also observed that Talc and colloidal silicon dioxide (Aerosil) do not have any significant effect on adhesion time and percent hydration, even at 5% concentration (fig. 1).

**Fig. 1:**
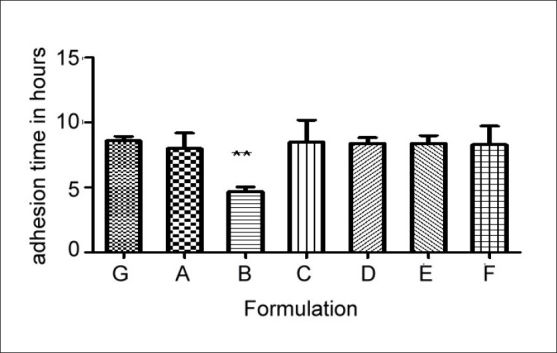
Effect of flow aids on mucoadhesion. One way ANOVA test was applied to adhesion time of different formulations. **Indicates p < 0.01 which means there is significant effect of excipient on adhesion time of polymer in case of Formulation B.

In practice, lubricants are used in a concentration (0.5-5%)[1]. According to the observations of this study, magnesium stearate has deleterious effects on mucoadhesion at a concentration >2%. Therefore, it is desirable to use talc and colloidal silicon dioxide (Aerosil) in place of magnesium stearate in formulation of Mucoadhesive Drug Delivery System (MDDS).
